# Evaluation of deltoid muscle quality in reverse shoulder arthroplasty: correlation of MRI findings with postoperative function and strength

**DOI:** 10.1007/s00330-025-11836-2

**Published:** 2025-07-19

**Authors:** Balázs Bogner, Arsenij Molotkov, Martin Jaeger, Andreas Hupperich, Ferdinand C. Wagner, Maximilian T. Löffler, Ralph Strecker, Reto Sutter, Fabian Bamberg, Hagen Schmal, Thierno D. Diallo, Pia M. Jungmann, Matthias Jung

**Affiliations:** 1 Department of Diagnostic and Interventional Radiology, University Medical Center Freiburg, Faculty of Medicine, University of Freiburg, Freiburg, Germany; 2 Berta-Ottenstein-Programme, Faculty of Medicine, University of Freiburg, Freiburg, Germany; 3 Department of Trauma Surgery, University Medical Center Munich, Faculty of Medicine, Technical University Munich, Munich, Germany; 4 Department of Orthopedic and Trauma Surgery, University Medical Center Freiburg, Faculty of Medicine, University of Freiburg, Freiburg, Germany; 5 EMEA Scientific Partnerships, Siemens Healthcare GmbH, Erlangen, Germany; 6 Department of Radiology, Balgrist University Hospital, Faculty of Medicine, University of Zurich, Zurich, Switzerland

**Keywords:** Shoulder joint, Total shoulder replacement, Deltoid muscle, Biomarkers, Magnetic resonance imaging

## Abstract

**Objectives:**

To evaluate associations between MRI-derived deltoid muscle parameters (areal fatty infiltration [FI] and cross-sectional area [CSA]) and clinical outcomes one year after reverse total shoulder arthroplasty (RTSA).

**Materials and methods:**

In this prospective, cross-sectional study, RTSA patients underwent 1.5-T MRI one year after surgery. Deltoid areal FI (%) and CSA (normalized to height, [CSAnorm, mm²/m]) were quantified in anterior, lateral, and posterior parts on T2-weighted imaging with metal artifact reduction. Spearman’s correlation coefficients and age- and sex-adjusted linear regression models assessed associations with clinical outcomes (Constant-Murley Score [CMS] and isometric strength [N]).

**Results:**

Among 25 patients (75 years [IQR 70–79], 72% females, BMI 28.7 kg/m² [IQR 24.6–29.4]) whole deltoid CSAnorm was positively correlated with CMS (*r* = 0.51, *p* = 0.010) and abduction strength (*r* = 0.48, *p* = 0.015), while FI showed negative correlations (*r* = −0.46 to 0.60, *p* = 0.002–0.019). Anterior CSAnorm positively correlated with CMS (*r* = 0.55, *p* = 0.005) and strength (*r* = 0.41 to −0.42, *p* = 0.034-0.040), while anterior FI showed negative correlations with CMS (*r* = −0.79, *p* < 0.001) and strength (*r* = −0.58 to −0.63, *p* = 0.002–0.003). Lateral CSAnorm correlated with CMS (*r* = 0.49, *p* = 0.014) but not with strength, while lateral FI showed no significant correlations. After adjustment, higher anterior CSAnorm was associated with better function and strength (β = 0.84–1.14, *p* = 0.003–0.028), and higher FI was associated with worse function and strength (β = −0.49 to −0.92, *p* < 0.001–0.014). Lateral CSAnorm remained associated with CMS (β = 0.40, *p* = 0.034), while lateral FI showed no significant associations.

**Conclusions:**

Metal artifact-reduced MRI-derived FI and CSAnorm are associated with shoulder function and strength one year after RTSA, potentially serving as objective markers for postoperative assessment.

**Key Points:**

***Question*** *RTSA success is measured by functional outcomes, but the relationship between postoperative imaging findings and functional results remains unclear*.

***Findings*** *Lower CSA and increased FI of deltoid, particularly anterior, were associated with reduced function and strength after RTSA*.

***Clinical relevance*** *Metal artifact-reduced MRI can assess postoperative deltoid FI and CSA, providing objective imaging biomarkers that complement clinical functional testing in the evaluation of patients following RTSA*.

**Graphical Abstract:**

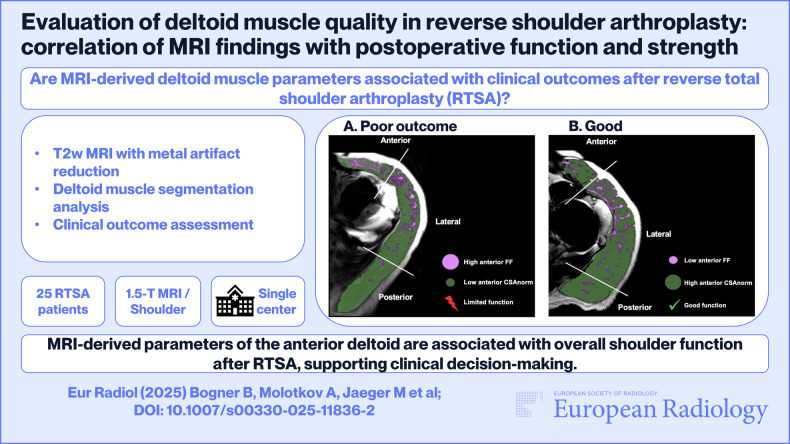

## Introduction

Implantation rates of reverse total shoulder arthroplasty (RTSA) have almost tripled from 2011 to 2017 in the US [1], driven by aging populations and expanding indications for RTSA. While RTSA was initially used to treat patients with irreparable rotator cuff tears and subsequent cuff arthropathy [2, 3], it is now recommended for various pathologies, including glenohumeral arthritis with severe glenoid deformity, complex proximal humerus fractures, revision shoulder arthroplasty, and bone tumors [4–7]. Overall, postoperative results have become more satisfactory as surgeons have gained more experience with the RTSA [8]. However, in patients with suboptimal outcomes after RTSA, the underlying contributing factors are often unclear [9–11]. Potential factors associated with poorer outcomes include inferior scapular notching, lever arm lengthening, and patient-related factors such as age and sex [12–15]. In addition, there is increasing evidence that deltoid muscle quality plays a crucial role in achieving optimal shoulder function after RTSA [16–18]. As RTSA medializes the center of rotation of the glenohumeral joint and extends the deltoid lever arm, it enhances deltoid muscle fiber recruitment. This makes the deltoid the primary muscle for shoulder flexion and abduction [16, 19].

Magnetic resonance imaging (MRI) allows for non-invasive qualitative and quantitative assessment of muscle integrity. MRI-derived muscle integrity parameters include muscle size, typically measured as cross-sectional area (CSA), and fatty infiltration (FI) [20, 21]. CSA serves as a surrogate for muscle strength and function, while increased FI indicates muscle degeneration. Previous studies showed an association between preoperative deltoid muscle parameters and postoperative outcomes after RTSA [22–25]. However, the clinical use of MRI in postoperative muscle evaluation in patients with suboptimal outcomes has been limited due to extensive susceptibility artifacts around the metal implant [26]. Metal artifact reduction MRI to visualize periprosthetic soft tissue is established for hip arthroplasty [27, 28]. However, imaging RTSA remains challenging due to the shoulder joint’s eccentric location and limited soft tissue coverage [29]. To date, only one study has evaluated the deltoid muscle after RTSA and reported a positive correlation between deltoid CSA and strength [30].

In this prospective study, we comprehensively assessed deltoid muscle integrity one year after RTSA using a T2-weighted MR imaging sequence with view angle tilting (VAT) for metal artifact reduction. We investigated the association between deltoid muscle FI, CSA, and shoulder function and strength.

## Materials and methods

This study was approved by the local Institutional Review Board (EK-Freiburg: 334/19) and registered prospectively at the German Clinical Trials Register (Trial registration number: DRKS00020807). All procedures were performed according to the ethical standards of the institutional and national research committee and to the Declaration of Helsinki in its current form. Informed written consent was obtained from all study participants.

### Study population

All patients underwent RTSA for severe rotator cuff deficiency between December 2019 and February 2022. The implants used were Medacta Reverse (Medacta International SA; *n* = 14), Arthrex Univers Revers^TM^ (Arthrex GmbH; *n* = 6), and Mathys Affinis Inverse (Mathys AG; *n* = 5). All procedures were performed by a single fellowship-trained, board-certified orthopedic surgeon using a standard deltopectoral approach (M.J., 20 years of experience). Postoperatively, all patients followed the same rehabilitation protocol. During the first three weeks, patients wore a 15° shoulder abduction cushion in neutral rotation, with glenohumeral motion restricted to 90° abduction. From week four onwards, the orthosis was discontinued, and abduction beyond 90° was permitted. One year after RTSA, patients were prospectively recruited for a single postoperative MRI scan during routine consultations in our Department of Orthopedic Surgery, independent of any clinical complaints. Inclusion criteria were: age > 18 years. Exclusion criteria were: MR contraindications (e.g., pacemaker, cochlear implants, severe claustrophobia, and gunshot wounds) and pregnancy. Additionally, all participants underwent clinical examination and interview during orthopedic consultation prior to study enrollment. No participants presented with conditions that could potentially confound muscle signal characteristics due to excessive edema, such as acute infection or recent trauma.

### Shoulder function assessment

The shoulder function was assessed using the Constant-Murley Score (CMS), which ranges from 0 (worst function) to 100 (best function) [31]. The CMS includes four modules: pain (15 points), activities of daily living (20 points), active range of motion (40 points), and strength (25 points). The first two modules, pain and activities of daily living, were assessed through standardized questionnaires. Pain was evaluated on a 15-point visual analog scale, where 0 indicates severe pain and 15 indicates no pain.

A score between 86 and 100 points represents a “very good” result, 71–85 points a “good” result, 56–70 points a “fair” result, and below 56 points is considered a “poor” result [32].

**Fig. 1:**
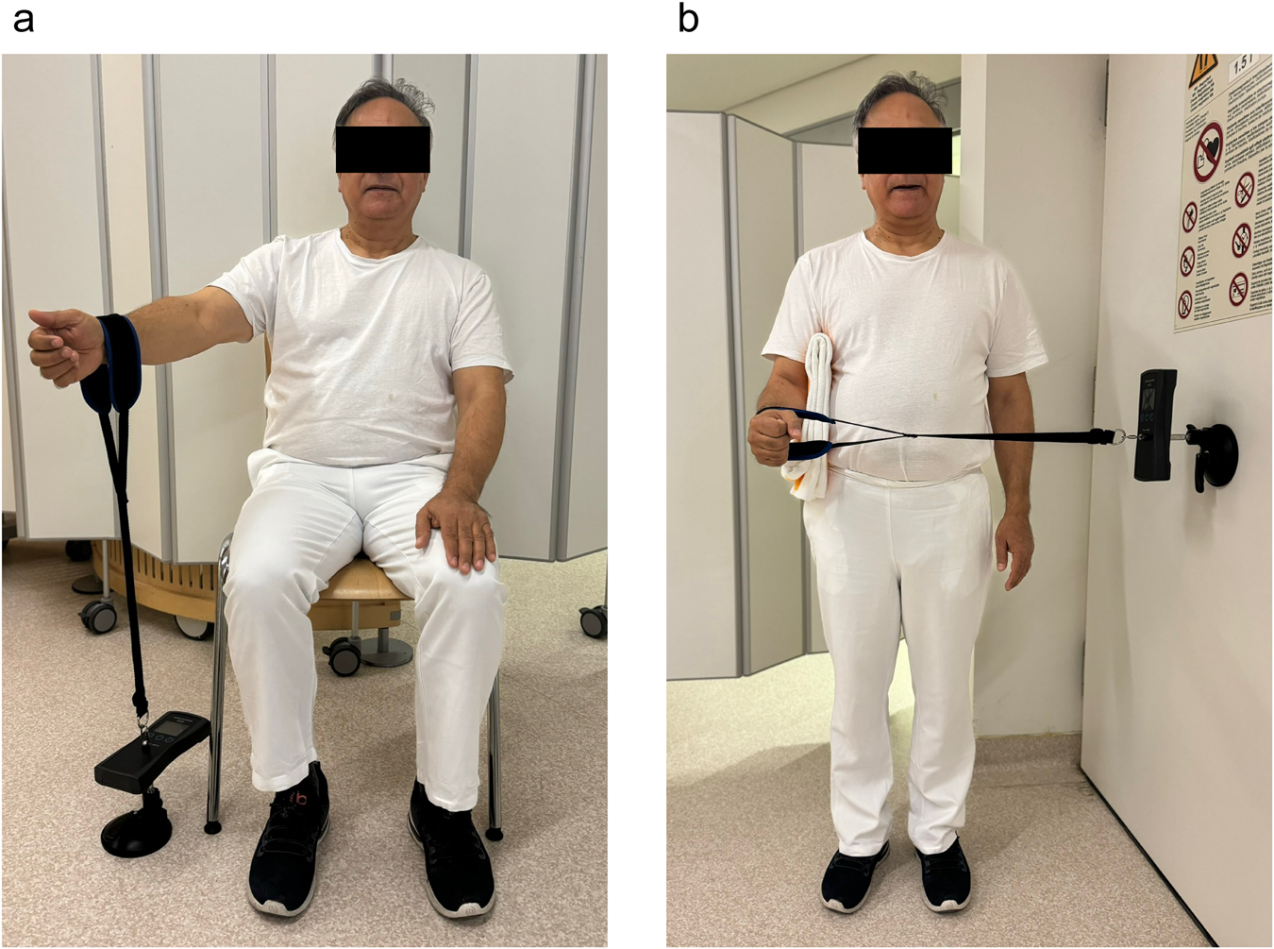
Clinical measurement of isometric strength of the right shoulder using a dedicated dynamometer was demonstrated on a healthy volunteer. **a** Abduction strength measurement in Jobe’s test position and (**b**) external rotation strength measurement

Activities of daily living were assessed based on the ability to perform specific tasks, including sleep, work, recreation/sport, and positioning the hand at different levels. Active range of motion was evaluated using a goniometer to measure forward flexion, abduction, external rotation, and internal rotation. Isometric strength (in Newton, N) was measured using an isometric dynamometer (IsoForceControl, Herkules Kunststoff AG). The abduction strength was measured in *Jobe’s test position*. Subjects were seated, and the arm was elevated at 90° in the scapular plane and externally rotated at 45° (Fig. 1a). The external rotation strength was measured in a standing position, and the flexed elbow at 90° (Fig. 1b). Mean isometric strength was used for subsequent data analysis.

### MRI acquisition

MRI of the operated shoulder was performed on a 1.5-T scanner (MAGNETOM Avanto Fit, Siemens Healthineers), using an 18-channel body coil (Siemens Healthineers). An axial two-dimensional T2-weighted (T2w) turbo spin-echo sequence, optimized for metal artifact reduction including VAT (syngo WARP in Siemens; repetition time [TR] = 4500 ms, echo time [TE] = 103 ms, slices = 30, flip angle = 170°, voxel size = 0.5 × 0.5 × 4.0 mm, phase encoding direction = anterior-posterior, field of view = 200 × 200 mm, bandwidth = 501 Hz/pixel, VAT = 50%, PAT mode = none, averages = 1, whole acquisition time = 1:45 min:s) and a coronal two-dimensional T1-weighted (T1w) turbo spin-echo sequence with WARP metal artifact reduction (repetition time [TR] = 489 ms, echo time [TE] = 8.6 ms, slices = 30, flip angle = 150°, voxel size = 0.5 × 0.5 × 3.0 mm, phase encoding direction = feet-head, field of view = 240 × 240 mm, bandwidth = 514 Hz/pixel, VAT = 50%, PAT mode = GRAPPA, acceleration factor PE = 2, averages = 1, whole acquisition time = 2:11 min:s) were acquired.

### Initial image evaluation

All images were initially reviewed before proceeding to qualitative and quantitative assessment. The metal artifact reduction technique proved sufficient for image analysis in all included cases, allowing complete evaluation of the deltoid muscle. The Goutallier score of the whole deltoid muscle was determined by a board-certified radiologist with 10 years of experience in musculoskeletal radiology (T.D.D.) according to Goutallier et al [33]. Semiquantitative MRI analysis was performed on a web-based medical image viewer (Nora Medical Imaging Platform; available free of charge online: http://ukl-nora-demo.ukl.uni-freiburg.de/nora/index.php?viewer).

### Anatomical landmark selection

**Fig. 2:**
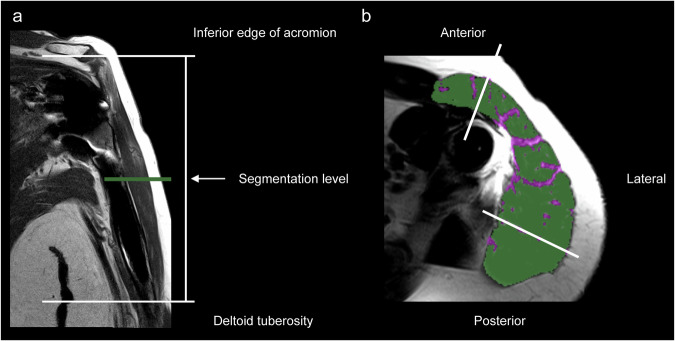
Deltoid muscle segmentation method. **a** Coronal T1w MRI sequence showing the selected measurement level (green line) for axial analysis at the mid-level between the inferior edge of the acromion and deltoid tuberosity. **b** Corresponding axial T2w sequence with WARP metal artifact reduction demonstrating segmentation of lean deltoid muscle (green overlay) and intramuscular fat (purple overlay), with anatomical subdivision into anterior, lateral, and posterior parts. Note that despite metal artifact reduction, susceptibility artifacts are still visible around the implant components, but do not significantly affect the deltoid muscle analysis at this level

First, the deltoid tuberosity and the inferior edge of the acromion were identified on the coronal T1w turbo spin-echo sequences. Then, the measurement level for muscle analysis was defined at the mid-level between these two anatomical landmarks (Fig. 2a), following a previously described approach [30], but relying solely on MRI for landmark identification.

### Deltoid muscle segmentation and quantitative measurements

Segmentation was performed on axial T2-weighted turbo spin-echo sequences with WARP metal artifact reduction. First, T2w sequences were individually windowed to depict sharp borders between epimysial (= perimuscluar) and perimysial (= intermuscular) connective tissue/fat (high signal intensity) and fascicles of the deltoid muscle (low signal intensity), creating a binary appearance of individual bundles of muscle fibers and their surrounding connective tissue. T2w images were visually compared to coronal T1w sequences to exclude bias due to muscle edema. Second, a mask outlining the entire muscle comprising all fascicles was manually defined based on the previous contrast window. Third, semi-automated region-growing segmentation was performed to mask individual fascicles within the previously defined muscle outline. The lean CSA in mm² of the deltoid muscle was calculated based on this mask. To account for the patient’s body size, the deltoid lean muscle CSA was normalized to the patient’s height:

$${{\rm{CSAnorm}}}({{{\rm{mm}}}}^{2}/{{\rm{m}}})=\frac{{{\rm{Deltoid}}}\; {{\rm{lean}}}\; {{\rm{CSA}}}({{{\rm{mm}}}}^{2})}{{{\rm{Patient}}}\; {{\rm{height}}}({{\rm{m}}})}$$

The intramuscular fat area in mm^2^ was calculated as the difference between the area of the entire deltoid muscle and the lean CSA. The deltoid FI was defined as described before [24]:

$$	{{\rm{Areal}}}\; {{\rm{fatty}}}\; {{\rm{infiltration}}}({{\rm{FI}}},\, \% )=\\ 	 \frac{{{\rm{Intramuscular}}}\; {{\rm{fat}}}\; {{\rm{area}}}({{{\rm{mm}}}}^{2})}{\,\left({{\rm{Intramuscular}}}\; {{\rm{fat}}}\; {{\rm{area}}}\left[{{{\rm{mm}}}}^{2}\right]+{{\rm{Lean}}}\; {{\rm{CSA}}}\left[{{{\rm{mm}}}}^{2}\right]\right)}$$

Individual thresholds for the region growing algorithm in the third step of our approach varied due to differences in MR signal intensity (caused by magnetic field inhomogeneities around the metallic implant and eccentric shoulder positioning, and potential muscle edema). For the entire cohort, the mean of the lower threshold and upper threshold were 115.2 ± 22.2 and 205.1 ± 40.1, respectively, where voxels below the lower threshold were classified as muscle tissue and voxels above the upper threshold were classified as intramuscular fat (Fig. 2b) shows the segmentation approach of the deltoid muscle.

### Image analysis workflow

One radiologist with 5 years of experience (B.B.) and one medical student with 3 years of experience (A.M.) performed segmentations independently on axial T2w images of the entire dataset, also reviewing the coronal T1w sequences to minimize bias from muscle edema. The medical student received previous training from a musculoskeletal radiologist with 10 years of experience (T.D.D.) focusing on the muscular anatomy of the upper extremity. To assess intra-observer agreement, measurements were repeated by the primary reader (B.B.) after a minimum of four weeks to ensure retest independence. The expert reader (T.D.D.) reviewed all segmentations on a case-by-case basis, blinded to reader identity, and selected one of the available segmentations (from B.B.’s initial reading, B.B.’s repeat reading, or A.M.’s reading) as the final segmentation, incorporating information from coronal T1w sequences. In four cases, minor adjustments were made to the selected segmentation. This process established the final segmentation dataset used for all primary analyses throughout the study. Intraclass correlation coefficients (ICCs) were calculated to assess inter-observer reliability between the two initial readers and intra-observer reliability for the repeat measurements.

### Deltoid anatomy classification

The deltoid was divided into three parts (Fig. 2b): anterior (clavicular), lateral (acromial), and posterior (spinous). These parts were identified by their distinct tendinous origins - the anterior part from the clavicle and the anterior surface of the acromion, the lateral part from the lateral aspect of the acromion, and the posterior part from the scapular spine [34]. Throughout this manuscript, these are referred to as the anterior, lateral, and posterior deltoid.

### Statistical analysis

To evaluate the intra- and inter-observer agreement of the image segmentation results, we calculated the ICCs (two-way mixed-effects analysis of variance, ANOVA). The following scale was used to interpret the ICC results: poor (ICC < 0.5), moderate (ICC = 0.5–0.7), good (ICC = 0.7–0.9), or excellent (ICC > 0.9) reproducibility [35]. Visual assessment in quantile-quantile plots showed a non-normal distribution for most parameters. Therefore, the data is presented as median and interquartile range (IQR). Differences in FI between deltoid parts (anterior, lateral, and posterior) were evaluated using the Friedman test, with post-hoc Wilcoxon signed-rank tests and Bonferroni correction for multiple comparisons (adjusted significance level *p* < 0.017). Spearman correlation coefficients (*r*) were calculated to assess the monotonic relationship between FI, CSAnorm, and clinical outcome parameters. The associations between FI, CSAnorm, and clinical outcome parameters were assessed in univariable and multivariable linear regression models adjusted for age and sex. Although multiple statistical tests were performed, we did not apply formal corrections for multiple testing. This should be taken into account when interpreting the significance of our results.

*p*-values < 0.05 were considered to be statistically significant. All statistical analyses were performed using R statistics (R-4.2.2 – R Core Team, https://www.r-project.org/).

## Results

### Intraobserver and interobserver agreement of image segmentation

Our segmentation approach demonstrated excellent reliability. Interreader reliability showed ICC = 0.930 (95% CI: 0.849–0.969) for FI and ICC = 0.953 (95% CI: 0.891–0.979) for CSA measurements. Intrareader reliability was similarly excellent with ICC = 0.951 (95% CI: 0.882–0.979) for FI and ICC = 0.929 (95% CI: 0.845–0.968) for CSA measurements.

### Baseline characteristics

A total of 25 individuals (18 females) with a median age of 75.0 years (IQR = 70.0–79.0) and body mass index (BMI) of 28.7 kg/m² (IQR = 24.6–29.4) were included in this study. The shoulder function assessed by CMS was 75.0 (IQR = 65.0–82.0). Isometric strength measurements revealed an abduction strength of 43 N (IQR = 37–61) and an external rotation strength of 37 N (IQR = 28–56).

The whole deltoid muscle had a CSAnorm of 131.1 mm²/m (IQR = 124.2–157.0) with a FI of 11.1% (IQR = 8.4–15.7). Analysis of deltoid subdivisions revealed regional differences in muscle characteristics. The anterior part showed a CSAnorm of 24.3 mm²/m (IQR = 14.9–29.4) and FI of 9.6% (IQR = 5.6–23.0), while the lateral part had a CSAnorm of 67.1 mm²/m (IQR = 54.4–89.7) and FI of 13.8% (IQR = 12.1–17.7). The posterior part demonstrated a CSAnorm of 41.7 mm²/m (IQR = 27.6–55.5) and FI of 4.4% (IQR = 2.8–7.9).

The Friedman test revealed significant differences in FI between deltoid parts (*p* < 0.001). Post-hoc analysis showed that the posterior deltoid had significantly lower FI compared to both anterior (*p* = 0.005) and lateral parts (*p* < 0.001), while no significant difference was found between anterior and lateral deltoid FI (*p* = 0.26). The Goutallier scores in the whole deltoid muscle ranged from grade 1 to 3, with the majority (*n* = 17; 68%) classified as grade 2, while six patients (24%) were graded as grade 1 and two patients (8%) as grade 3. Baseline characteristics are summarized in Table 1.

**Table 1:** Baseline characteristics, median (IQR) or proportion (%)

Characteristic	Value
Number of subjects	25
Age (years)	75.0 (70.0–79.0)
Sex (female)	18 (72%)
BMI (kg/m^2^)	28.7 (24.6–29.4)
CMS	75.0 (65.0–82.0)
Abduction strength (N)	43 (37–61)
External rotation strength (N)	37 (28–56)
Whole deltoid lean CSA (mm^2^)	229.3 (209.9–261.3)
Whole deltoid CSAnorm (mm^2^/m)^†^	131.1 (124.2–157.0)
Whole deltoid FI (%)	11.1 (8.4–15.7)
Anterior deltoid lean CSA (mm^2^)	40.9 (26.6–49.2)
Anterior deltoid CSAnorm (mm^2^/m)^†^	24.3 (14.9–29.4)
Anterior deltoid FI (%)	9.6 (5.6–23.0)
Lateral deltoid lean CSA (mm^2^)	108.9 (91.9–148.7)
Lateral deltoid CSAnorm (mm^2^/m)^†^	67.1 (54.4–89.7)
Lateral deltoid FI (%)	13.8 (12.1–17.7)
Posterior deltoid lean CSA (mm^2^)	67.8 (49.0–95.7)
Posterior deltoid CSAnorm (mm^2^/m)^†^	41.7 (27.6–55.5)
Posterior deltoid FI (%)	4.4 (2.8–7.9)
Goutallier score	
1	6 (24%)
2	17 (68%)
3	2 (8%)

*BMI* body mass index, *CMS* constant-Murley score, *CSA* cross-sectional area, *CSAnorm* normalized cross-sectional area, *FI* fatty infiltration

^†^ Lean CSA normalized to patient height

### Correlations between deltoid CSAnorm, FI, and clinical outcome parameters

Whole deltoid CSAnorm showed moderate positive correlations with CMS (*r* = 0.51, *p* = 0.010) and abduction strength (*r* = 0.48, *p* = 0.015) but not with external rotation strength (*r* = 0.30, *p* = 0.150). Whole deltoid FI demonstrated moderate negative correlations with CMS (*r* = −0.60, *p* = 0.002), abduction strength (*r* = −0.46, *p* = 0.019), and external rotation strength (*r* = −0.58, *p* = 0.003). No significant correlations were found between Goutallier scores and CMS (*r* = −0.07, *p* = 0.740), abduction strength (*r* = 0.01, *p* = 0.970), or external rotation strength (*r* = −0.002, *p* = 0.990).

For the anterior deltoid, CSAnorm demonstrated moderate positive correlations with CMS (*r* = 0.55, *p* = 0.005), abduction strength (*r* = 0.41, *p* = 0.040), and external rotation strength (*r* = 0.42, *p* = 0.034). The anterior deltoid FI showed strong negative correlations with CMS (*r* = −0.79, *p* < 0.001), abduction strength (*r* = −0.63, *p* < 0.001), and moderate negative correlations with external rotation strength (*r* = −0.58, *p* = 0.002).

For the lateral deltoid, CSAnorm demonstrated a moderate positive correlation with CMS (*r* = 0.49, *p* = 0.014), while no significant correlations were found with strength measurements (*r* = 0.25–0.27, all *p* > 0.05, Table 2). FI of the lateral deltoid showed no significant correlations with shoulder function (*r* = −0.13, *p* = 0.550) or strength measurements (*r* = −0.13 to −0.25, all *p* > 0.05, Table 2). Non-significant correlations of the posterior muscle integrity parameters and clinical outcome measures are presented in Table 2.

**Table 2:** Spearman correlations between deltoid CSAnorm, FI, and clinical outcome parameters

	CMS		Abduction strength, N		External rotation strength, N	
	*r*	*p*	*r*	*p*	*r*	*p*
Whole deltoid						
CSAnorm, mm^2^/m	0.51	**0.010**	0.48	**0.015**	0.30	0.150
FI, %	−0.60	**0.002**	−0.46	**0.019**	−0.58	**0.003**
Anterior deltoid						
CSAnorm, mm^2^/m	0.55	**0.005**	0.41	**0.040**	0.42	**0.034**
FI, %	−0.79	**< 0.001**	−0.63	**< 0.001**	−0.58	**0.002**
Lateral deltoid						
CSAnorm, mm^2^/m	0.49	**0.014**	0.25	0.220	0.27	0.190
FI, %	−0.13	0.550	−0.13	0.540	−0.25	0.230
Posterior deltoid						
CSAnorm, mm^2^/m	0.011	0.960	0.17	0.420	0.0073	0.970
FI, %	−0.0092	0.970	0.14	0.510	0.11	0.610

Bold indicates statistical significance

*CMS* constant-Murley score, *CSAnorm* height-normalized cross-sectional area, *FI* fatty infiltration, *r* Spearman’s correlation coefficient

**Fig. 3:**
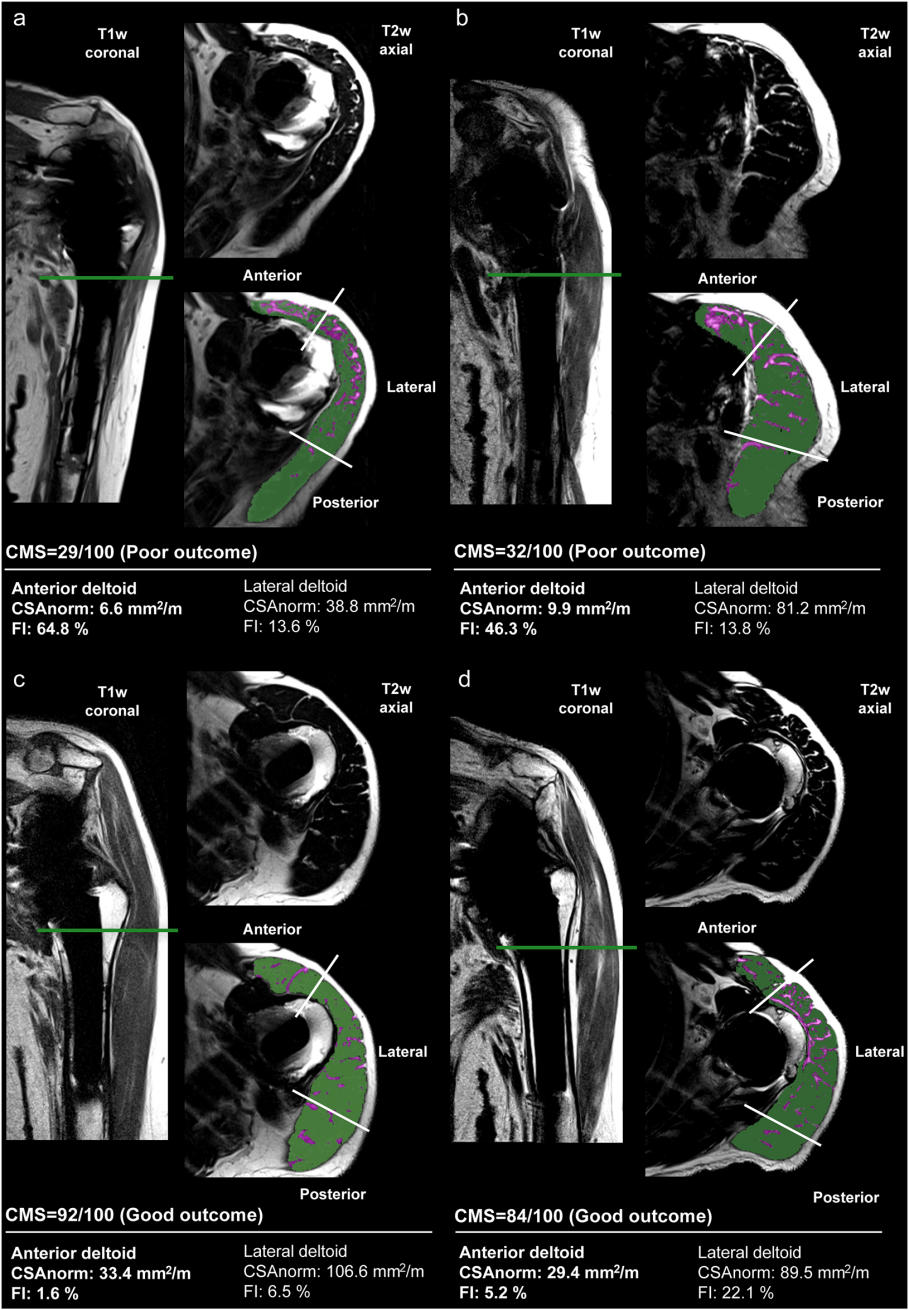
Representative cases of poor and good clinical outcomes after RTSA are demonstrated by T1w coronal and T2w axial MRI sequences with WARP metal artifact reduction and corresponding segmentation overlay. **a**, **b** Poor outcome cases exhibit markedly higher anterior deltoid FI compared to (**c**, **d**) good outcome cases. Note that good functional outcomes can occur despite moderate lateral deltoid FI. MRI-derived deltoid muscle parameters for the anterior and lateral deltoid are provided below each case. In all cases, the left shoulder is featured. CSAnorm, cross-sectional area normalized to patient height; FI, fatty infiltration; CMS, constant-Murley score; WARP, Siemens sequence for metal artifact reduction, including VAT

Representative cases illustrating the relationship between deltoid muscle parameters and clinical outcomes are shown in Fig. 3. Poor outcome cases (Fig. 3a, b) demonstrate higher FI (64.8% and 46.3%) with reduced CSAnorm (6.6 mm²/m and 9.9 mm²/m) of the anterior deltoid, corresponding to inferior clinical outcomes with lower CMS (29/100 and 32/100), decreased abduction strength (24 N and 38 N) and external rotation strength (28 N and 28 N). In contrast, good outcome cases (Fig. 3c, d) show substantially lower FI (1.6% and 5.2%) with higher CSAnorm (33.4 mm²/m and 29.4 mm²/m) of the anterior deltoid, corresponding to better clinical outcomes with higher CMS (92/100 and 84/100), greater abduction strength (68 and 70 N) and external rotation strength (56 and 70 N). Notably, good clinical outcomes can be achieved despite elevated lateral deltoid FI (22.1%), as demonstrated in Fig. 3d.

### Univariable linear regression models

In univariable linear regression models, increased whole deltoid CSAnorm was significantly associated with higher CMS (β = 0.38, 95% CI [0.14, 0.61], *p* = 0.003) and abduction strength (β = 0.30, 95% CI [0.05, 0.55], *p* = 0.020).

Increased whole deltoid FI was significantly associated with lower CMS (β = −1.20, 95% CI [−2.27, −0.13], *p* = 0.029), lower abduction strength (β = −1.31, 95% CI [−2.34, −0.29] *p* = 0.014) and external rotation strength (β = −1.21, 95% CI [−2.20, −0.22], *p* = 0.019).

The anterior deltoid CSAnorm showed significant positive associations with CMS (β = 1.10, 95% CI [0.43, 1.78], *p* = 0.003), abduction strength (β = 0.77, 95% CI [0.03, 1.52], *p* = 0.042), and external rotation strength (β = 0.87, 95% CI [0.19, 1.56], *p* = 0.015). The anterior deltoid FI showed significant negative associations with CMS (β = −0.92, 95% CI [−1.17, −0.68], *p* < 0.001), abduction strength (β = −0.59, 95% CI [−0.98, −0.20], *p* = 0.004), and external rotation strength (β = −0.50, 95% CI [−0.89, −0.12], *p* = 0.013).

Increased lateral deltoid CSAnorm showed significant associations with higher CMS (β = 0.34, 95% CI [0.01, 0.68], *p* = 0.043**)**, while no significant associations were found with strength measurements (β = 0.19 to 0.28, all *p* > 0.05, Table 3). FI of the lateral deltoid showed no significant associations with CMS or strength measurements (β = −0.48 to 0.02, all *p* > 0.05, Table 3).

**Table 3:** Associations between deltoid CSAnorm, FI, and clinical outcome parameters

Region and predictor	Model	CMS		Abduction strength, N		External rotation strength, N	
		β (95% CI)	*p*	β (95% CI)	*p*	β (95% CI)	*p*
Whole deltoid							
CSAnorm, mm^2^/m	Unadj	0.38 (0.14, 0.61)	**0.003**	0.30 (0.05, 0.55)	**0.020**	0.24 (–0.01, 0.49)	0.055
	Adj	0.43 (0.18, 0.68)	**0.002**	0.37 (0.12, 0.62)	**0.005**	0.26 (0.005, 0.05)	**0.046**
FI, %	Unadj	–1.20 (–2.27, –0.13)	**0.029**	–1.31 (–2.34, –0.29)	**0.014**	–1.21 (–2.20, –0.22)	**0.019**
	Adj	–1.35 (–2.63, –0.07)	**0.040**	–1.21 (–2.44, 0.02)	0.054	–1.08 (–2.26, 0.09)	0.069
Anterior deltoid							
CSAnorm, mm^2^/m	Unadj	1.10 (0.43, 1.78)	**0.003**	0.77 (0.03, 1.52)	**0.042**	0.87 (0.19, 1.56)	**0.015**
	Adj	1.14 (0.44, 1.85)	**0.003**	0.84 (0.10, 1.58)	**0.028**	0.88 (0.21, 1.56)	**0.013**
FI, %	Unadj	–0.92 (–1.17, –0.68)	**< 0.001**	–0.59 (–0.98, –0.20)	**0.004**	–0.50 (–0.89, –0.12)	**0.013**
	Adj	–0.92 (–1.18, –0.66)	**< 0.001**	–0.57 (–0.96, –0.18)	**0.006**	–0.49 (–0.87, –0.11)	**0.014**
Lateral deltoid							
CSAnorm, mm^2^/m	Unadj	0.34 (0.01, 0.68)	**0.043**	0.19 (–0.16, 0.54)	0.268	0.28 (–0.05, 0.60)	0.090
	Adj	0.40 (0.03, 0.78)	**0.034**	0.24 (–0.13, 0.62)	0.194	0.27 (–0.08, 0.62)	0.126
FI, %	Unadj	0.02 (–0.87, 0.91)	0.963	–0.48 (–1.33, 0.37)	0.258	–0.41 (–1.24, 0.41)	0.309
	Adj	0.17 (–0.86, 1.21)	0.734	–0.28 (–1.25, 0.70)	0.562	–0.16 (–1.09, 0.76)	0.716

Beta coefficients (β) are from unadjusted (Unadj) and age- and sex-adjusted (Adj) linear regression models. They represent the increase in CMS, abduction strength, or external rotation strength for a unit increase in CSAnorm or FF. Bold indicates statistical significance

*CMS* constant-Murley score from 0 to 100 (= best function), *CSAnorm* height-normalized cross-sectional area, *FI* fatty infiltration, *CI* confidence interval

### Multivariable linear regression models

After adjusting for age and sex in linear regression models, increased whole deltoid CSAnorm remained significantly associated with higher CMS (β = 0.43, 95% CI [0.18, 0.68], *p* = 0.002), abduction strength (β = 0.37, 95% CI [0.12, 0.62], *p* = 0.005), and external rotation strength (β = 0.26, 95% CI [0.005, 0.05], *p* = 0.046). Increased whole deltoid FI was significantly associated with lower CMS (β = −1.35, 95% CI [−2.63, −0.07], *p* = 0.040) but not with strength measurements (β = −1.21 to −1.08, all *p* > 0.05, Table 3).

Increased anterior deltoid CSAnorm showed significant associations with higher CMS (β = 1.14, 95% CI [0.44, 1.85], *p* = 0.003), abduction strength (β = 0.84, 95% CI [0.10, 1.58], *p* = 0.028), and external rotation strength (β = 0.88, 95% CI [0.21, 1.56], *p* = 0.013).

Increased anterior deltoid FI remained significantly associated with lower CMS (β = −0.92, 95% CI [−1.18, −0.66], *p* < 0.001), abduction strength (β = −0.57, 95% CI [−0.96, −0.18], *p* = 0.006), and external rotation strength (β = −0.49, 95% CI [−0.87, −0.11], *p* = 0.014).

Increased lateral deltoid CSAnorm remained a significant predictor of higher CMS (β = 0.40, 95% CI [0.03, 0.78], *p* = 0.034). FI of the lateral deltoid showed no significant associations with CMS or strength measurements (β = −0.28 to 0.17, all *p* > 0.05, Table 3).

## Discussion

This study investigated the association between MRI-derived parameters of deltoid muscle integrity, shoulder function, and strength one year after RTSA. Our main findings showed that normalized whole deltoid muscle CSA was positively associated, and areal FI was negatively associated with overall shoulder function after surgery. Of note, CSA and FI of the anterior deltoid had the strongest effect on shoulder function. These findings highlight the importance of deltoid muscle integrity after RTSA and indicate differential contributions of deltoid segments to postoperative function.

RTSA has emerged as the primary surgical approach for patients with cuff arthropathy [2, 3]. It causes significant biomechanical changes in the shoulder joint, resulting in the deltoid becoming the primary motor muscle for arm motion and joint stability [16, 19, 36, 37]. Preoperative muscle size has been identified as a significant predictor of clinical outcomes after RTSA [23, 24]. In addition, increased preoperative FI in the deltoid has been associated with inferior postoperative results [17, 24]. However, little is known about the association between postoperative deltoid muscle properties and shoulder function. Metal artifact reduction MRI sequences have been successfully applied to the postoperative evaluation of hip and knee prostheses [27, 38]. After arthroplasty at the shoulder, imaging is challenging due to the shoulder joint’s eccentric position and limited soft tissue coverage. Therefore, MRI studies investigating postoperative RTSA outcomes are scarce. To date, only one study investigated postoperative deltoid properties and found a positive correlation between deltoid muscle size and shoulder function [30]. In line with these findings, the present study showed that a higher CSAnorm of the deltoid muscle was associated with better CMS, abduction, and external rotation strength one year after RTSA. In addition, increased deltoid FI showed significant associations with worse clinical outcomes.

Previous studies reported distinct biomechanical changes in the different anatomical parts (anterior, lateral, and posterior) of the deltoid after RTSA [19, 39]; in particular, the anterior deltoid plays a crucial role in optimal shoulder function [40], and anterior deltoid rupture has been reported to result in poorer functional outcomes [41]. A cadaver study using biomechanical measurements confirmed the clinical observations that the anterior deltoid is vital for optimal shoulder function after RTSA [19]. In line, we found strong associations between anterior deltoid CSAnorm, FI, and clinical outcome measures. Our findings further support the hypothesis that anterior deltoid integrity may be key for optimal outcomes following RTSA.

Besides the anterior part, the lateral deltoid is a relevant shoulder abductor after RTSA, and we found a significant positive association between CSAnorm and shoulder function for the lateral deltoid [42]. While postoperative degeneration of the lateral deltoid has been described, its impact on clinical outcomes remains controversial [17, 24]. In this study, lateral deltoid FI showed no associations with shoulder function and strength one year after RTSA, suggesting that degenerative changes of the lateral deltoid may not significantly impact postoperative outcomes. The medialization of the center of rotation after RTSA optimizes the anterior deltoid’s lever arm, while the relatively short lateral deltoid may be lengthened beyond its optimal length-tension relationship. These biomechanical changes result in optimal shoulder function relying predominantly on the anterior rather than the lateral deltoid [24].

Regarding the posterior deltoid, biomechanical studies have demonstrated its role in external rotation, particularly in patients with deficient teres minor and infraspinatus muscles [43]. Interestingly, our study found no significant correlations between posterior deltoid parameters and external rotation strength. Instead, we observed correlations between anterior deltoid CSAnorm and FI and external rotation strength measurements. These findings might be explained by a functional interplay between the anterior deltoid and the remaining teres minor and infraspinatus muscles [43].

Our study has several limitations. First, due to our small sample size, subgroup analyses comparing different implant designs or stratifying by varying degrees of degenerative changes in the remaining rotator cuff were not feasible. Current literature indicates that the FI of the teres minor and infraspinatus muscles impacts functional outcomes [44], and different implant designs lead to differential patterns of degenerative changes in the remaining rotator cuff [45]. These factors potentially confounded the associations between deltoid muscle parameters and functional outcomes. Future studies should include larger cohorts in multicenter settings to analyze this complex biomechanical interplay.

Second, the absence of baseline imaging limited our ability to determine whether the observed muscle characteristics were present before surgery or developed afterward. Similarly, without preoperative functional assessment, we cannot quantify the degree of functional change following surgery. This temporal gap limits our assessment of causality, whether superior deltoid muscle quality leads to improved outcomes, or whether improved functional outcomes contribute to better muscle maintenance. It also hampers our ability to differentiate natural anatomical variations from the effects of the surgical approach on different deltoid segments.

Finally, our image analysis had methodological limitations. We used a semi-quantitative approach on T2w MRI with metal artifact reduction for muscle-fat segmentation. Although our segmentations of deltoid FI were carefully validated with T1w sequences, muscle edema signals may potentially affect our FI measurements [46]. Despite recent advancements in metal artifact reduction techniques, quantitative water-fat imaging using the Dixon technique remains unfeasible due to severe artifacts in the periprosthetic region [47]. Additionally, while we employed a semi-automated segmentation technique, the process still required manual corrections, introducing a potential degree of subjectivity.

In conclusion, our findings highlight the importance of postoperative MRI-derived deltoid muscle parameters (CSAnorm and FI) one year after RTSA. While postoperative FI of the lateral deltoid did not have clinical relevance, the integrity of the anterior deltoid, in particular, may be crucial for optimal shoulder function. Therefore, postoperative MRI assessment of muscle parameters may provide valuable insight in those patients with poorer outcomes after RTSA and help guide subsequent clinical decision making and therapeutic interventions.

## Abbreviations

BMI: Body mass index
CMS: Constant-Murley score
CSA: Cross-sectional area
FI: Fatty infiltration
ICC: Intraclass correlation coefficient
IQR: Interquartile range
MRI: Magnetic resonance imaging
N: Newton
RTSA: Reverse total shoulder arthroplasty
VAT: View angle tilting
